# Eplerenone in the treatment of central serous chorioretinopathy: a review of the literature

**DOI:** 10.1186/s40942-018-0137-8

**Published:** 2018-09-19

**Authors:** Irini Chatziralli, Aikaterini Vlachodimitropoulou, Chrysoula Daoula, Christina Vrettou, Eleni Galani, George Theodossiadis, Panagiotis Theodossiadis

**Affiliations:** 1 2nd Department of Ophthalmology, National and Kapodistrian University of Athens, 28, Papanastasiou Street, Agios Dimitrios, 17342 Athens, Greece; 2 Medical School, National and Kapodistrian University of Athens, Athens, Greece

**Keywords:** Central serous retinopathy, Eplerenone, Chronic, Mileralocorticoid

## Abstract

**Purpose:**

The purpose of this review is to examine the role of eplerenone in the treatment of central serous chorioretinopathy (CSCR).

**Methods:**

A comprehensive search of the PubMed database has been conducted regarding eplerenone for CSCR, while studies using spironolactone were excluded. Articles and book chapters cited in the reference lists of articles obtained by this method were reviewed and included when considered appropriate, while the retrieved articles were filtered manually to exclude duplicates.

**Results:**

Oral eplerenone at a dose of 25–50 mg/day has been found to be effective and well-tolerated for the treatment of chronic CSCR. The published studies have shown significant improvement in visual acuity and decrease or total absorption of subretinal fluid in patients with CSCR treated with oral eplerenone. However, it should be noted that the majority of studies were retrospective with limited number of patients and short follow-up. On the other hand, patients presenting widespread retinal pigment epithelium changes are less likely to benefit from eplerenone treatment, which may argue for an earlier intervention.

**Conclusions:**

CSCR is a challenging disease to understand and treat, since its pathogenesis remains elusive and multifactorial. Pharmacologic approaches, like eplerenone, are intriguing, as they target several pathophysiological pathways and may lead to visual acuity improvement and more rapid recovery.

## Background

Central serous chorioretinopathy (CSCR) is a chorioretinal disease, characterized by serous detachment of the neurosensory retina and/or retinal pigment epithelium (RPE) with consequent accumulation of fluid [1–3]. In fact, the location and amount of fluid determines the symptomatology in patients with CSCR. If the fluid is located outside the macula, there may be no symptoms, while if the detachment affects the central macula, symptoms may include visual acuity decrease, metamorphopsia, changes in image size, decrease in contrast sensitivity, perception of blind spots or combination of these symptoms [1]. The diagnosis is performed by dilated fundus exam and imaging of the retina and the choroid with optical coherence tomography, fluorescein angiography and indocyanine green angiography [4, 5].

CSCR is a common cause of visual impairment in the working-age population and has been estimated as the fourth most frequently encountered non-surgical retinopathy after age-related macular degeneration, diabetic retinopathy and retinal vein occlusion [1–3]. It typically affects young to middle-aged men (30–50 years old), but patients with chronic disease (duration more than 6 months) may continue to suffer from the disease even in advanced age [1–3]. In a population-based study conducted in Minnesota, the reported annual incidence of CSCR was 9.9 per 100,000 male cases compared to 1.7 per 100,000 women [6]. Apart from gender differences, it seems that there is ethnic predilection with Asians presenting higher incidence compared to other ethnic populations [7].

Although the exact pathogenesis of CSCR remains elusive, a number of risk factors for this disorder have been implicated. High levels of endogenous (i.e., in Cushing’s syndrome or in pregnancy) or exogenous (i.e., intra-articular, intranasal, systemic or topical) corticosteroids, type A personality, obstructive sleep apnea, abnormal coagulation and platelet aggregation, infection with Helicobacter pylori, male gender, pregnancy, smoking, hypertension, antibiotic use, alcohol consumption and oxidative stress have been considered to be the most significant risk factors for the development of CSCR [5, 8–10]. Moreover, genetic susceptibility seems to play an important role in the pathophysiology of CSCR and genetic polymorphisms have been associated with CSCR [11–13].

The disease is usually idiopathic and often resolves spontaneously with visual recovery, although occasionally neurosensory retinal detachment persists or relapses and leads to permanent damage of the RPE and photoreceptors with subsequent visual impairment [1–3]. Current treatment modalities for CSCR generally target the RPE, choroid, or both. They aim to improve the ability of the RPE to remove the subretinal fluid, to diminish leakage from the choroidal vessels, or to decrease fluid flux across the RPE barrier [4, 5]. Management usually involves either waiting for spontaneous resolution, which commonly occurs within 3 months of onset, or the use of focal laser photocoagulation, photodynamic therapy with verteporfin and anti-vascular endothelial growth factor (anti-VEGF) agents in cases of choroidal neovascularization related to CSCR [4]. Recently, mineralocorticoid receptors (MR) have been implicated in the pathophysiology of CSCR; therefore, factors targeting these receptors may be used for the treatment of CSCR [14]. In light of the above, the purpose of this review is to examine the role of eplerenone, an MR antagonist, in the treatment of CSCR.

## Literature search

We conducted a comprehensive search of the PubMed database to include articles up to December 31th, 2017, using the following search algorithm: (central serous retinopathy OR central serous chorioretinopathy) AND (eplerenone OR mineralocorticoid). Only studies or cases series evaluating patients with CSCR treated with eplerenone were included in this review, while studies regarding treatment with spironolactone were excluded. Articles and book chapters cited in the reference lists of articles obtained by this method were reviewed and included when considered appropriate, while the retrieved articles were filtered manually to exclude duplicates.

### Pathophysiology of CSCR and the “mineralocorticoid receptor” theory

The pathogenesis of CSCR is multifactorial and incompletely understood, making its treatment challenging. The first theory, proposed by Gass, suggested that there is a focal choroidal hyper-permeability, leading to leakage of fluid into the subretinal space [15]. However, Marmor claimed that a focal disruption of the RPE could not cause serous detachment owing to the ability of RPE cells to compensate, and he proposed that CSCR is the result of diffuse metabolic impairment of the RPE [16]. In addition, choroidal ischemia has been shown to be involved in choroidal hyper-permeability and RPE dysfunction [17].

Recently, significant progress has been made on the understanding of the pathogenesis of CSCR, regarding the molecular events triggering choroidal vasodilatation in CSCR. Noticeably, CSCR is the only retinal disease involving fluid accumulation, which is not improved but even worsened by corticosteroids [18]. Corticosteroids include both glucocorticoid (cortisol) and mineralocorticoid (aldosterone), while receptors for glucocorticoid and mineralocorticoid are expressed in Mueller cells and choroidal vessels [18, 19]. Zhao et al. have found that systemic and local glucocorticoids, which are known risk factors for CSCR, act by binding both to the receptor for glucocorticoid and that for mineralocorticoid with equally high affinity [20]. Additionally, Daruich et al. proposed that over activation of the MR in the choroidal endothelial cells induces upregulation of the vasodilator potassium channel KCa2.3, which modulates smooth muscle cells relaxation in the choroidal vasculature [21, 22]. This process has been shown to cause choroidal vasodilation, fluid accumulation in the retina, and to promote retinal neovascularization in hypoxic conditions [18]. Therefore, this link between corticosteroids and CSCR, combined with the observation of an induced CSCR-like model in the rat following MR pathway activation, has prompted the evaluation of MR antagonist in the treatment of CSCR [14].

### Eplerenone in the treatment of CSCR

Eplerenone is an MR antagonist with an increased MR selectivity and higher affinity compared to spironolactone. However, eplerenone has about 10- to 20- fold lower binding to progesterone and androgen receptors and does not include the hormonal effects to the same extent, thereby limiting sex-hormone-related adverse side effects [23, 24]. Zhao et al. first reported the treatment of two patients with chronic CSCR using oral eplerenone, presenting rapid subretinal fluid resolution and improvement in visual acuity, which was maintained at 5 months after cessation of treatment [14]. Since then, several studies have investigated the efficacy and safety of eplerenone for CSCR treatment [14, 21, 25–38], as it is depicted on Table 1. Summarizing the results of these studies, oral eplerenone at a dose of 25–50 mg/day has been found to be effective and well-tolerated for the treatment of chronic CSCR. There was no significant difference between the two dosages, although it should be noted that the follow-up of the studies is short-term and no randomized study has compared the two treatment regimens [35–37]. In addition, the majority of studies were retrospective with limited number of patients [14, 26–34]. Sampo et al. conducted a retrospective study with the largest study sample of 27 patients with chronic CSCR and demonstrated statistically significant anatomical and functional improvement in such patients at the 3-month follow-up [34]. Prospective studies have shown similar results [25, 35–38]. Only two prospective, randomized studies were published, comparing eplerenone treatment to placebo and showing statistically significant improvement in visual acuity and decrease in subretinal fluid height, as well as central macular thickness, although both of them presented short follow-up time (9 weeks and 6 months respectively) [36, 38]. On the other hand, patients presenting widespread RPE changes are less likely to benefit from eplerenone treatment, which may argue for an earlier intervention [33]. Apart from studies and case series, two case reports have been published in the literature [39, 40], showing similar results with those of above-mentioned studies. Nevertheless, it has to be mentioned that eplerenone did not influence the hyperfluorescent pattern, which is seen in patients with chronic CSCR on fluorescein angiography and indocyanine green angiography [23].

**Table 1:** Characteristics and results of studies evaluating oral eplerenone for the treatment of central serous chorioretinopathy

Study	Study design	N eyes	Disease duration	Visual acuity change	Subretinal fluid height change	Central macular thickness change	Follow-up	Side-effects
Schwartz (2017) [38]	Prospective, randomized	13	Chronic	Improvement from 0.6 to 0.48 logMAR (p = 0.05)	Improvement from 143.3 to 101.7 μm (p = 0.021)	NA	6 months	Increased CPK
Pichi (2017) [37]	Prospective study	20	Chronic	Improvement from 0.2 to 0 logMAR (p = 0.03)	Improvement from 247 to 35 μm (p = 0.004)	NA	2 months	Sedative effect and fatigue
Rahimy (2017) [36]	Prospective, randomized controlled study	15	Chronic	Improvement from 0.394 ± 0.28 to 0.330 ± 0.27 logMAR (p = 0.04)	Improvement from 139.3 ± 58.7 to 51.8 ± 52.2 μm (p = 0.02)	Improvement from 366.2 ± 71.1 to 283.7 ± 65.4 μm (p = 0.02)	9 weeks	Dizziness, diarrhea
Gergely (2017) [35]	Prospective	28	Chronic	Improvement from 75.1 to 78.1 letters (p < 0.005)	Improvement from 207 to 120 μm (p < 0.005)	Improvement from 393 to 324 μm (p < 0.005)	6 months	Dry mouth, dizziness, back pain, somnolence
Sampo (2016) [34]	Retrospective series	27	Chronic	Improvement from 0.26 to 0.19 logMAR (p = 0.15)	Decrease of 93.04 μm (p = 0.00018)	Improvement from 371.6 to 294.3 μm (p = 0.038)	3 months	Hyperkaliemia
Cakir (2016) [33]	Retrospective series	24	Chronic	Improvement from 0.35 to 0.3 logMAR	Improvement from 117 to 65 μm	Improvement from 342 to 275 μm	3 months	Myotonia, bowel irritation, hyperkaliemia
Kapoor (2016) [32]	Retrospective series	12	Chronic	Improvement from 0.55 to 0.32 logMAR (p < 0.05)	ΝΑ	Improvement from 324.7 to 259.6 μm (p < 0.05)	3 months	Fatigue, weight loss, gynecomastia
Ghadiali (2016) [31]	Retrospective series	3	2 Chronic, 1 Acute	Improvement from 0.67 to 0.75 decimal scale (p = 0.043)	ΝΑ	Improvement from 310.3 to 304.7 μm (p = 0.125)	12 months	Hypertension
Leisser (2015) [30]	Retrospective series	11	Chronic	Change in visual acuity from 0.48 to 0.71 logMAR	ΝΑ	Improvement from 455 to 389 μm	3-38 weeks	Increased liver parameters, Increased potassium, Increase bilirubin level
Chin (2015) [29]	Retrospective series	15	Chronic	Stability at 20/30 Snellen	NA	Improvement from 387.5 to 352.5 μm	NA	Fatigue, leg cramps, constipation, dehydration
Salz (2015) [28]	Retrospective series	14	Chronic	Improvement from 0.41 to 0.28 logMAR (p = 0.01)	Improvement from 130 to 21 μm (p = 0.004)	NA	3 months	None
Singh (2015) [27]	Retrospective series	17	Chronic	Improvement from 0.42 to 0.29 logMAR (p = 0.024)	Improvement from 131.5 to 46.9 μm (p = 0.002)	Improvement from 339.5 to 270.3 μm (p = 0.029)	6 months	None
Breukink (2014) [26]	Retrospective series	6	Chronic	Improved in 2 patients	Decreased in 2 patients	NA	5 weeks	None
Bousquet (2013) [25]	Prospective, non-randomized uncontrolled open label study	13	Chronic	Improvement from 0.52 ± 0.24 to 0.27 ± 0.19 logMAR (p < 0.001)	Improvement from 175 ± 123 to 36 ± 55 μm (p < 0.01)	Improvement from 352 ± 139 to 189 ± 99 μm (p < 0.01)	3 months	Fatigue, sedative effect
Zhao (2012) [14]	Retrospective series	3	Chronic	Improvement from 0.43 to 0.8 decimal scale	Decreased in all eyes	NA	5 months	None

*NA* non applicable

Potential adverse events of eplerenone may include hyperkalemia, which can be exaggerated by coexisted renal insufficiency, diabetes mellitus, advanced heart failure, older patient age and interactions with other medications, such as potassium-sparing diuretics, angiotensin converting enzyme inhibitors and angiotensin receptor blockers, fatigue, dizziness, sedative effect, hypertension, diarrhea or constipation, bowel irritation, myotonia, gynecomastia, weight loss and increase of serum liver parameters, bilirubin and CPK levels [25–38]. However, adverse events seem to be dose-dependent and are reversible after discontinuation of treatment.

It is worthy to note that since most of the studies examining eplerenone for CSCR treatment are retrospective with short-term follow-up and small sample size, large prospective, randomized studies are needed to scrutinize the role of MR antagonists in CSCR treatment.

## Conclusions

CSCR is a challenging disease to understand and treat, since its pathogenesis remains elusive and multifactorial. Systemic emerging pharmacologic approaches, like eplerenone, are intriguing, as they target several pathophysiological pathways and may lead to visual acuity improvement and more rapid recovery. Based on the current literature, eplerenone seems to be efficient, especially at the chronic stage of the disease. Since the results on patients with widespread RPE changes are limited and non-significant, further research is needed to determine which patients are most likely to benefit from eplerenone and their imaging characteristics, while potential combination with other treatment modalities can be also considered.
